# Asymmetric temperature effect on leaf senescence and its control on ecosystem productivity

**DOI:** 10.1093/pnasnexus/pgae477

**Published:** 2024-10-23

**Authors:** Lei He, Jian Wang, Josep Peñuelas, Constantin M Zohner, Thomas W Crowther, Yongshuo Fu, Wenxin Zhang, Jingfeng Xiao, Zhihua Liu, Xufeng Wang, Jia-Hao Li, Xiaojun Li, Shouzhang Peng, Yaowen Xie, Jian-Sheng Ye, Chenghu Zhou, Zhao-Liang Li

**Affiliations:** State Key Laboratory of Efficient Utilization of Arid and Semi-arid Arable Land in Northern China, Institute of Agricultural Resources and Regional Planning, Chinese Academy of Agricultural Sciences, Beijing 100081, China; College of Earth and Environment Sciences, Lanzhou University, Lanzhou 730000, China; The Key Laboratory of Land Surface Pattern and Simulation, Institute of Geographical Sciences and Natural Resources Research, Chinese Academy of Sciences, Beijing 100101, China; University of the Chinese Academy of Sciences, Beijing 100049, China; CSIC, Global Ecology Unit, CREAF- CSIC-UAB, Cerdanyola del Vallès, 08193 Barcelona, Catalonia, Spain; CREAF, Cerdanyola del Vallès, 08193 Barcelona, Catalonia, Spain; Institute of Integrative Biology, ETH Zurich (Swiss Federal Institute of Technology), 8092 Zurich, Switzerland; Institute of Integrative Biology, ETH Zurich (Swiss Federal Institute of Technology), 8092 Zurich, Switzerland; College of Water Sciences, Beijing Normal University, Beijing 100875, China; Department of Physical Geography and Ecosystem Science, Lund University, Lund 22362, Sweden; Earth Systems Research Center, Institute for the Study of Earth, Oceans, and Space, University of New Hampshire, Durham, NH 03824, USA; CAS Key Laboratory of Forest Ecology and Management, Institute of Applied Ecology, Chinese Academy of Sciences, Shenyang 110016, China; Key Laboratory of Remote Sensing of Gansu Province, Heihe Remote Sensing Experimental Research Station, Northwest Institute of Eco-Environment and Resources, Chinese Academy of Sciences, Lanzhou 730000, China; State Key Laboratory of Resources and Environmental Information System, Institute of Geographic Sciences and Natural Resources Research, Chinese Academy of Sciences, Beijing 100101, China; INRAE, UMR1391 ISPA, Université de Bordeaux, Villenave d'Ornon 33140, France; State Key Laboratory of Soil Erosion and Dryland Farming on the Loess Plateau, Northwest A&F University, Yangling 712100, China; College of Earth and Environment Sciences, Lanzhou University, Lanzhou 730000, China; Key Laboratory of Western China's Environmental Systems (Ministry of Education), Lanzhou University, Lanzhou 730000, China; State Key Laboratory of Herbage Improvement and Grassland Agro-Ecosystems, College of Ecology, Lanzhou University, Lanzhou 730000, China; Center for Ocean Remote Sensing of Southern Marine Science and Engineering Guangdong Laboratory (Guangzhou), Guangzhou Institute of Geography, Guangdong Academy of Sciences, Guangzhou 510070, China; State Key Laboratory of Efficient Utilization of Arid and Semi-arid Arable Land in Northern China, Institute of Agricultural Resources and Regional Planning, Chinese Academy of Agricultural Sciences, Beijing 100081, China; State Key Laboratory of Resources and Environmental Information System, Institute of Geographic Sciences and Natural Resources Research, Chinese Academy of Sciences, Beijing 100101, China

## Abstract

Widespread autumn cooling occurred in the northern hemisphere (NH) during the period 2004–2018, primarily due to the strengthening of the Pacific Decadal Oscillation and Siberian High. Yet, while there has been considerable focus on the warming impacts, the effects of natural cooling on autumn leaf senescence and plant productivity have been largely overlooked. This gap in knowledge hinders our understanding of how vegetation adapts and acclimates to complex climate change. In this study, we utilize over 36,000 in situ phenological time series from 11,138 European sites dating back to the 1950s, and 30 years of satellite greenness data (1989–2018), to demonstrate that leaf senescence dates (LSD) in northern forests responded more strongly to warming than to cooling in autumn. Specifically, a 1 °C increase in temperature caused 7.5 ± 0.2 days' delay in LSD, whereas a 1 °C decrease led to an advance of LSD with 3.3 ± 0.1 days (*P* < 0.001). This asymmetry in temperature effects on LSD is attributed to greater preoverwintering plant-resource acquisition requirements, lower frost risk, and greater water availability under warming than cooling conditions. These differential LSD responses highlight the nonlinear impact of temperature on autumn plant productivity, which current process-oriented models fail to accurately capture. Our findings emphasize the need to account for the asymmetric effects of warming and cooling on leaf senescence in model projections and in understanding vegetation–climate feedback mechanisms.

**Significance Statement**

Autumn cooling was widespread in the northern hemisphere from 2004 to 2018; however, its influence on leaf senescence and plant productivity remains poorly understood. Using in situ observations, satellite data, and model estimates, we demonstrate that leaf senescence in northern forests responds more significantly to natural autumn warming than cooling. This study highlights the association between the asymmetric responses of leaf senescence and plant productivity to temperature changes and contributes to the improvement of model projections.

## Introduction

The timing of autumn leaf senescence plays a critical role in the regulation of carbon (C) and nutrient cycling in terrestrial ecosystems (1–5). Understanding the changes in leaf senescence dates (LSD) under climate change is therefore essential for the accurate prediction of future dynamics and feedbacks of global biogeochemical cycles (6). Existing studies have identified varied responses of LSD to climate warming, leading to divergent patterns of LSD (delayed, advanced, or stable) (7–9). Compared with spring phenology, a detailed understanding of autumn phenological dynamics remains lacking (10, 11), where the underlying mechanisms of complex LSD responses to temperature change are unclear (12).

Variability in LSD is driven by multiple climate and biotic factors, including temperature, precipitation, drought, photoperiod, wind speed, spring leaf-out date, and growing-season C assimilation (8, 13–20). Autumn temperatures exert direct effects on leaf physiological status, such as chlorophyll levels, photosynthesis, pigment degradation, and nutrient remobilization, ultimately triggering leaf senescence (21–23). Autumn temperatures may also exert indirect impacts on LSD through altering soil moisture and vapor pressure deficit (VPD) (24–26).

Although understanding of ecological effects of regional cooling under on-going global warming has increasingly gained attention (26–29), patterns of LSD responses to autumn warming and cooling scenarios remain a subject of ongoing debate that limits understanding of climate change impacts and maintains bias in forecasts of vegetation–climate interactions (30–32). For example, empirical field data show a stronger LSD response in European deciduous tree species to warmer than cooler autumn temperatures (30), whereas data from temperature manipulation experiments show larger LSD responses to cooling than to warming and a lack of difference between the two conditions (31, 32). Recent analyses indicate a substantial cooling trend in northern hemisphere (NH) autumn temperatures over the period 2004–2018 that is associated with the strengthening of Pacific Decadal Oscillation and Siberian High (26, 33); thus, long-term, large-scale data provide the opportunity to test for natural temperature-driven regulation and biophysical and biochemical drivers of autumn LSD and ecosystem C uptake.

To this end, we aim to quantify responses of autumn LSD and C update to natural warming and cooling and test for underlying mechanisms using ground and remotely sensed data, comprising a phenological time series of >36,000 records collected from 11,138 sites across Europe between 1951 and 2016 and estimates derived from normalized difference vegetation index (NDVI) data obtained between 1989 and 2018 covering the NH autumn cooling period at middle and high latitudes (>30°N). Specifically, we ask: (i) Does LSD in northern forests respond asymmetrically to natural autumn warming and cooling? (ii) What are the underlying mechanisms for these potential asymmetric patterns? (iii) Do asymmetrical temperature effects on LSD influence plant productivity responses?

### LSD responses to natural autumn warming and cooling

**Fig. 1.**
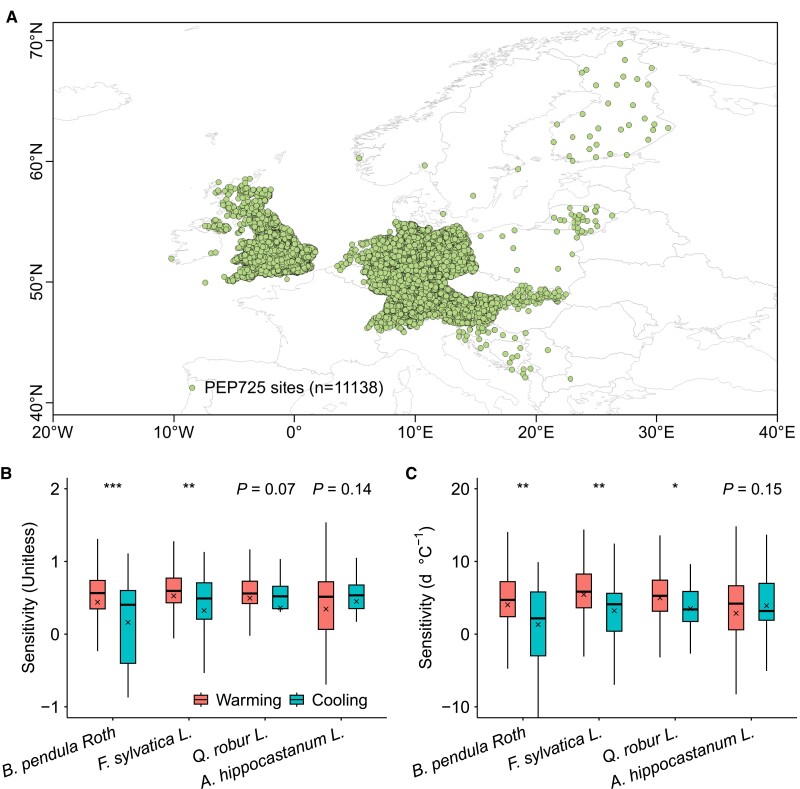
European distribution of study sites and analyses of LSD responses to natural autumn warming and cooling in *B. pendula Roth*, *F. sylvatica L.*, *Q. robur L.*, and *A. hippocastanum L*. A) Location of the long-term study site records for the four European temperate tree species. B) Ridge regression analyses of LSD responses. C) Multiple linear regression analyses of LSD responses. Records of warming and cooling were selected based on change in temperature and partial correlation analysis at *P* < 0.05 and differences in LSD responses between warming and cooling conditions were analyzed using Student's t test at *P* < 0.05. Boxplots show median (horizontal line) and mean (cross) data within the 25–75th percentiles; ***P < 0.001, **P < 0.01, and *P < 0.05.

At the species level, ridge and multiple linear regression analyses of in situ long-term records from the Pan European Phenology (PEP) database (34) (see Materials and methods) for four representative temperate tree species (Fig. 1A) indicated a tendency toward asymmetric LSD responses to natural autumn warming and cooling, evidenced by different changes in LSD caused by 1 °C increase. Apart from *Aesculus hippocastanum L.*, LSD's sensitivities to warming were overall greater than that to cooling in autumn for other three species, i.e. *Betula pendula Roth*, *Fagus sylvatica L.*, and *Quercus robur L.,* using ridge regression (Fig. 1B) and multiple linear regression analyses (Fig. 1C).

**Fig. 2.**
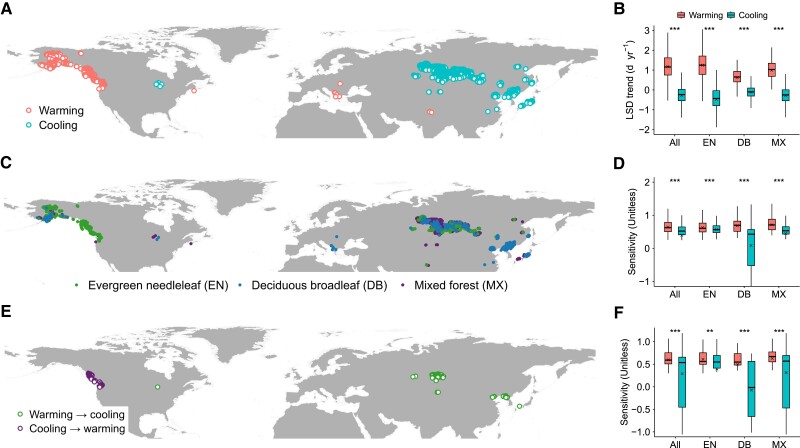
Grid cells of remotely sensed data in which there was autumn warming and cooling during the period (2004–2018) and analyses of forest biome LSD responses. A) Location of autumn warming and cooling grid cells. B) Trends in biome LSD. C) Location of forest biome types that experienced warming and cooling, based on the MODIS MCD12C1 IGBP land cover product. D) Ridge regression analysis of biome LSD sensitivity to temperature, controlling for effects of precipitation and radiation. E) Location of grid cells in which there were shifts in warming and cooling between the period 1989–2003 and 2004–2018. F) Ridge regression analysis of biome LSD sensitivity to temperature. Differences in LSD responses between warming and cooling conditions were analyzed using Student's t test at *P* < 0.05. Boxplots show median (horizontal line) and mean (cross) data within the 25–75th percentiles; ***P < 0.001, **P < 0.01, and *P < 0.05.

For the biome-level remote-sensing analyses, we applied a natural autumn cooling period (2004–2018) to identify warming and cooling sites (see Materials and methods) and test for LSD response patterns (Fig. 2). Results showed that LSD was delayed at warming sites and became earlier at cooling sites for evergreen needleleaf, deciduous broadleaf, and mixed forests (Fig. 2B), and the greater overall biome ridge sensitivity of LSD to autumn warming (0.64 ± 0.008, mean ± SE) than to cooling (0.39 ± 0.007) (*P* < 0.001) reflected consistent asymmetric LSD responses for evergreen needleleaf (0.63 ± 0.008 vs. 0.57 ± 0.009; *P* < 0.001), deciduous broadleaf (0.68 ± 0.04 vs. 0.09 ± 0.02; *P* < 0.001), and mixed forests (0.71 ± 0.02 vs. 0.45 ± 0.008; *P* < 0.001) (Fig. 2D). Similarly, multiple linear regression analyses showed asymmetric patterns of biome LSD responses to warming and cooling (all forests: 7.5 days/°C ± 0.2 vs. 3.3 ± 0.1, *P* < 0.001; evergreen needleleaf: 8.2 ± 0.2 vs. 4.8 ± 0.2, *P* < 0.001; deciduous broadleaf: 4.4 ± 0.3 vs. 2.3 ± 0.2, *P* < 0.001; mixed forests: 4.6 ± 0.3 vs. 3.3 ± 0.1, *P* < 0.001) (Fig. S1). Although we compared the LSD responses in each biome, the warming and cooling sites were spatially separated from each other, which might introduce uncertainty. Then, we conducted a temporal analysis to complement the spatial analyses, based on grid cells in which there were shifts in warming and cooling between the period 1989–2003 and 2004–2018 (see Materials and methods; Fig. 2E), and similarly found greater sensitivity of LSD responses to autumn warming than to cooling among the forest biomes (Fig. 2F and Fig. S2).

In line with a previous study of field observations of warm and cold autumn conditions identified from a long-term average temperature threshold (30), our analyses of ground and remotely sensed data showed larger tree species and forest biome LSD responses to autumn warming than to cooling, indicating nonlinear temperature control of leaf senescence; thus, cooling effects on LSD should not be assumed to be the inverse of those to warming (35). In contrast to our findings, temperature manipulation experiments show larger or null LSD responses to autumn cooling than to warming (31, 32), possibly due to confounding effects of unmeasured and/or uncontrollable environmental factors that lead to an underestimation of phenological responses in warming experiments (12, 36, 37).

### Mechanisms of asymmetric LSD responses to shifts in autumn temperature

We hypothesize that the asymmetric forest biome and species LSD responses to temperature shifts may be associated with differences in climatic forcing effects, such as cold degree days (CDD), adaptation to frost risk, and/or soil water availability (38, 39, 13). To test these hypotheses, we compared LSD responses to changes in CDD, preseason temperature variability and frost risk, and water stress, based on the Standardized Precipitation-Evapotranspiration Index (SPEI) at the autumn warming and cooling sites from 2004 to 2018 (see Materials and methods).

**Fig. 3.**
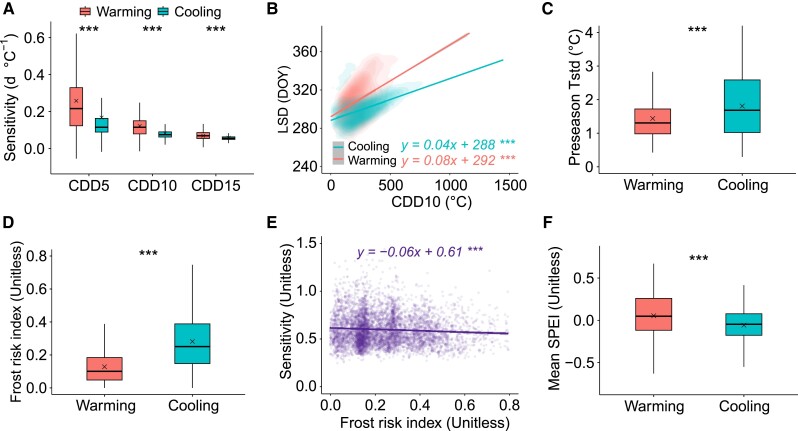
Mechanisms of asymmetric LSD responses to autumn warming and cooling. A) Sensitivities of LSD to CDD under autumn warming and cooling with contrasting CDD base temperatures of 5, 10, and 15 °C (CDD5, CDD10, and CDD15, respectively). B) Linear regression analysis of relations between LSD and CDD10 under autumn warming and cooling conditions; differences in regression slopes tested by covariance analysis at *P* < 0.001. C) Standard deviation in preseason temperature (Preseason Tstd) under autumn warming and cooling conditions. D) Mean frost risk index under autumn warming and cooling conditions. E) Linear regression analysis of relations between LSD sensitivity to temperature (ridge sensitivity absolute values) and frost risk index. F) Mean SPEI under autumn warming and cooling conditions. Differences between autumn warming and cooling conditions (A, C, D, F) were tested using a linear mixed method with random intercepts among forest biomes. Boxplots show median (horizontal line) and mean (cross) data within the 25–75th percentiles; ***P < 0.001, **P < 0.01, and *P < 0.05.

The first hypothesis accounts for changes in LSD response to exponential decreases in CDD with rising temperatures under warming and cooling conditions (Fig. S3), where sensitivities of LSD to CDD were larger under autumn warming than cooling, regardless of base temperature (5, 10, or 15 °C) for the calculation of CDD (*P* < 0.001) (Fig. 3A). Comparison of the relations between LSD and CDD under autumn warming and cooling and contrasting CDD base temperatures showed that LSD responses to CDD were larger under warming (*P* < 0.001) (Fig. 3B and Fig. S4), while random slope model estimates showed a larger LSD response to CDD under warming (*P* < 0.001), when effects of geographical location (latitude and longitude) were controlled (Fig. S5). Before leaf senescence, trees must absorb ample carbohydrate and nutrient stores to ensure tissue maturation, support overwintering and facilitate budburst in the subsequent spring (5, 30) and under favorable, autumn warming conditions, trees may delay leaf senescence to enhance C uptake (5, 30). Thus, our analyses indicate that under a given shift in CDD caused by warming or cooling, delays in leaf senescence caused by warming may be larger than advances caused by cooling, due to the asymmetry of LSD responses to changes in CDD.

Our second hypothesis relates to tree adaptations to frost damage (39–41), as indicated by studies that show spatial variation in phenology responses to temperature changes, with smaller responses observed at sites experiencing higher local temperature variability (40, 42). As expected, we found larger variations in temperature at cooling sites than sites that experienced warming, corresponding to lower LSD responses (Fig. 3C). Furthermore, the standardized frost risk index we developed to explore differences in frost risk between autumn warming and cooling conditions (See Materials and methods) showed greater risks of frost at cooling sites (Fig. 3D) that lead to conservative response strategies of leaf sensing to avoid frost damage, as indicated by decreased LSD responses under increasing risks of frost (Fig. 3E).

The third hypothesis concerns the potential effects of water stress on leaf senescence. We found that water stress levels were higher at the cooling sites than at the warming sites (Fig. 3F). This observation aligns with findings that water stress can significantly reduce leaf stomatal conductance and photosynthesis and enhance chlorophyll degradation (40, 43–45), potentially lowering the temperature sensitivity of leaf senescence at cooler locations. However, warming and more widespread droughts in the future (46–48) may increase the impact of droughts associated with warming on LSD (13). Consequently, this could result in a diminished LSD response to warming, akin to outcomes observed in temperature manipulation experiments that involve significant temperature variations (e.g. changes > 2 °C in warming and cooling scenarios) (32). This suggests a complex interplay between temperature, water availability, and their combined effects on leaf senescence, underlining the need for comprehensive studies to understand these dynamics.

### Links between autumn temperature shifts, LSD responses, and plant productivity

**Fig. 4.**
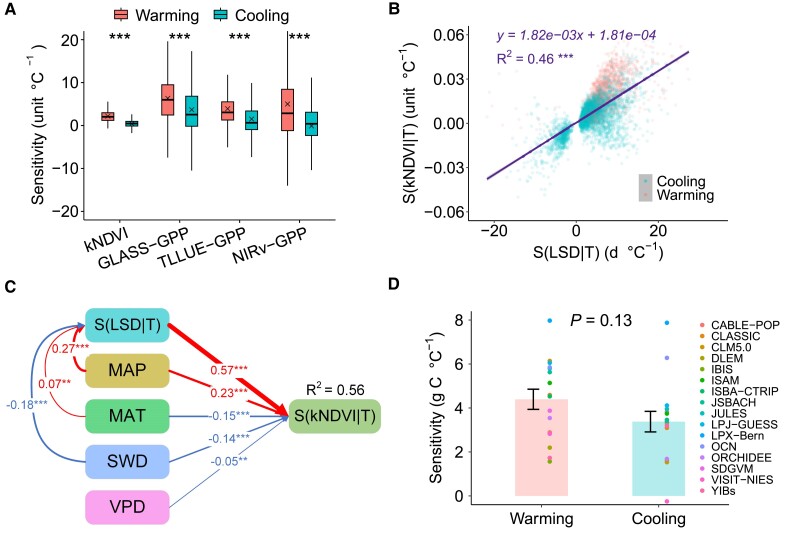
Responses and drivers of autumn productivity to warming and cooling. A) Sensitivities of autumn productivity to temperature under warming and cooling, based on kNDVI, GLASS-GPP, TLLUE-GPP, and NIRv-GPP data sets; kNDVI scaling factor is 0.01. B) Linear regression analysis of the relations between autumn kNDVI sensitivities to temperature [S(kNDVI|T)] and LSD sensitivities to temperature [S(LSD|T)]. C) PLS-SEM analysis of S(LSD|T) and climate factor effects on mean S(kNDVI|T); MAP, mean annual precipitation; MAT, mean annual temperature; SWD, downward solar radiation; VPD, vapor pressure deficit. D) Mean sensitivities of autumn GPP to temperature under warming and cooling in 16 DGVMs in the TRENDY-v11 project (see Materials and methods); sensitivity differences among models were tested using Student's t test at *P* < 0.05 and the bars indicate SE. ***P < 0.001, **P < 0.01.

Sensitivities of plant growth and productivity to temperature are highly variable across space and time and are regulated by a range of factors, such as vegetation type, prevailing climate, and temperature variability (49, 50). However, the lack of a mechanistic understanding of the drivers of the high levels of heterogeneity in sensitivities of plant productivity to temperature poses a challenge to the robustness of process-oriented models (50, 51). Indeed, we found the high levels of spatial heterogeneity in autumn productivity sensitivities to temperature across remotely sensed grid cells, as indicated by the climate space of mean annual precipitation (MAP) and mean annual temperature (MAT) (Fig. S6). Hence, this study analyzed the phenological temperature sensitivity of plant productivity and explored the contribution of asymmetric LSD responses to asymmetry in plant productivity under autumn warming and cooling. Four productivity datasets, comprising a kernel NDVI (kNDVI) and three gross primary productivity (GPP) datasets (Global Land Surface Satellite GPP, GLASS-GPP; two-leaf light use efficiency modeled GPP, TLLUE-GPP; near-infrared reflectance of vegetation-based GPP, NIRv-GPP), all lines of evidences confirmed a larger response of autumn productivity to increasing autumn temperatures than under cooling (Fig. 4A). Sensitivities of autumn productivity to temperature were positively correlated with LSD sensitivities to temperature, where LSD sensitivities accounted for 46% of spatial variability in autumn productivity sensitivities to temperature (Fig. 4B), while partial least-squares structural equation modeling (PLS-SEM) and random forest modeling analyses indicated that LSD sensitivity to temperature was the most important driver of autumn productivity sensitivity to temperature (Fig. 4C and Fig. S7). These results indicate that asymmetric LSD responses to autumn warming and cooling may lead to corresponding asymmetric responses in autumn productivity. If we do not account for the asymmetric productivity response to a 1 °C autumn temperature change (i.e. 1 °C warming or cooling), the biases in autumn plant productivity across the three studied forest biomes (i.e. evergreen needleleaf, deciduous broadleaf, and mixed forests) in the NH would amount to nearly 0.031 ± 0.007 Pg C yr^−1^ (Fig. S8; see Materials and methods), which is half the minimum value within the range of carbon loss from deforestation in the Amazon forest (0.06 to 0.21 Pg C yr^−1^) (52). As the magnitude of warming and cooling increases (e.g. 2 °C), the biases would increase further, indicating that asymmetric LSD and GPP responses to autumn warming and cooling significantly impact global terrestrial carbon budget.

We further analyzed sensitivities of autumn productivity to temperature under warming and cooling conditions using state-of-the-art Dynamic Global Vegetation Models (DGVMs) (see Materials and methods) and found that 11 out of 16 models showed larger responses of autumn productivity to warming compared with cooling (Fig. S9). The sensitivity comparison of the combined results from 16 models reveals a stronger, though not statistically significant, response to warming than to cooling (*P* = 0.13; Fig. 4D). These findings suggest that current state-of-the-art models partially but limitedly capture the observed differences in responses of autumn productivity to temperature changes under warming and cooling. One potential reason could be related to the complexity of phenological control and its climatic responses. Incorporating a detailed characterization of the asymmetric LSD responses may enhance the representation of asymmetric productivity responses to autumn warming and cooling in process-oriented models, thereby improving the accuracy of seasonal vegetation dynamics predictions.

## Conclusions

The phenological responses of trees to changing temperature will strongly regulate terrestrial carbon storage under climate change. By exploring the plant responses to warming vs. cooling, our study allows us to test for the presence of acclimation, which will underpin the extent and duration of phenological shifts under climate warming. Both in situ observations and satellite data suggest that the timing of LSD in northern forest biomes responds asymmetrically to natural variations in autumn temperatures, showing a more pronounced response to warming than to cooling. This disparity in LSD responses may be attributed to factors such as the greater sensitivity of LSD to CDD, lower risks of frost, and greater water availability under warming than cooling. Such asymmetric LSD reactions may, in turn, lead to unequal changes in autumn plant productivity in response to warming and cooling, a complexity that current DGVMs struggle to accurately represent. This new understanding is crucial for refining vegetation and climate models, thereby improving the accuracy of future carbon cycle and climate projections.

## Materials and methods

### In situ LSD observation

We used ground LSD observations, the date of 50% autumnal coloring of tree leaves (BBCH code 94), across Europe from the long-term plant phenological observation database, the PEP Project (PEP725, http://www.pep725.eu/) (34). We collected all available phenological time series (>36,000) from 11,138 study sites since the 1950s for four dominant tree species, comprising *A. hippocastanum L*., *B. pendula Roth*, *F. sylvatica L*., and *Q. robur L*., which had sufficient autumn warming and cooling samples (Fig. 1A). The median absolute deviation (MAD) method was applied to identify and remove erroneous data points in PEP725 records (30). For a time series of LSD_t1_, LSD_t2_, …, LSD_t_ at each site, the MAD is calculated as follows:


(1) $$\mathrm{MAD}=\mathrm{median}\;(|\mathrm{LS}D_{t}\;-\;\mathrm{median}\;(\mathrm{LSD})|)$$

The data point exceeding 2.5 times the MAD was deemed an outlier and consequently eliminated from the LSD time series before analysis.

### Remotely sensed greenness-derived LSD

We used the bi-weekly PKU Global Inventory Modeling and Mapping Studies (GIMMS) NDVI, at 1/12° spatial resolution, to estimate LSD between 1989 and 2018. The PKU GIMMS NDVI was produced utilizing a biome-specific back-propagation neural network algorithm that using GIMMS NDVI3g product and Landsat NDVI samples, and its temporal coverage was extended to 2022 following consolidation with the Moderate-Resolution Imaging Spectroradiometer (MODIS) NDVI using the random forest method. This new NDVI product efficiently eliminated the effects of sensor degradation and satellite orbital drift and showed high overall accuracy assessed by Landsat NDVI samples. To mitigate snow effects, we replaced all compromised NDVI values with the winter (December–February) mean of snow-free NDVI values across all years and then the NDVI time series was reconstructed using a Savitzky–Golay filter to eliminate abnormal values (53, 54); sparse vegetation cover was removed by eliminating grid cells with mean annual NDVI value < 0.1.

We estimated LSD using the double-logistic function and dynamic-threshold approaches, which were widely used methods for estimating phenological dates from remotely sensed data (13, 15, 28). A double-logistic function was fitted to the NDVI time series, followed by calculation of the second-order derivative for the fitted curve (55, 56), and LSD was determined as the date corresponding to the second local maximum in the second half year:


(2) $$y(t)=a+b\;\left(\frac{1}{1+e^{c(t\;-\;d)}}+\frac{1}{1+e^{e(t\;-\;f)}}\right)$$

where *y*(*t*) is the NDVI value at the day of year (DOY), *t*; *a* is the background NDVI value; and, *b–e* are the double-logistic function parameters.

For the dynamic-threshold approach, we computed NDVI ratio ($\mathrm{Rati}o_{\mathrm{day}}$) annually for each NDVI time series as follows (57):


(3) $$\mathrm{Rati}o_{\mathrm{day}}=\frac{\mathrm{NDV}I_{\mathrm{day}}\;\text{–}\;\mathrm{NDV}I_{\min}}{\mathrm{NDV}I_{\max}\;\text{–}\;\mathrm{NDV}I_{\min}}$$

where $\;\mathrm{NDV}I_{\min}$ and $\mathrm{NDV}I_{\max}$ are the minimum and maximum NDVI of each year, respectively, and $\mathrm{NDV}I_{\mathrm{day}}$ is the daily NDVI. LSD is determined as the time (DOY) when $\mathrm{Rati}o_{\mathrm{day}}$ decreased to 0.5 (57). The average LSD of the two approaches was calculated as the ultimate LSD.

### Plant productivity data

We used two types of plant productivity indicator, comprising the newly developed kNDVI and GPP; kNDVI is a widely used indicator of GPP (58) and it was calculated based on a simplified operational index version, which was expressed as $\mathrm{kNDVI}=\tanh(\mathrm{NDV}I^{2})$, using the monthly MODIS MOD13C2 v061 product (59). We used three independent GPP products to minimize uncertainty stemming from a single product: the eight-day GLASS GPP (60), the monthly TLLUE modeled GPP (61); and the monthly NIRv based GPP (62). All kNDVI/GPP data were resampled to 1/12° to match the spatial resolution of the PKU GIMMS NDVI.

### Process-oriented modeled GPP

We employed modeled GPP data from 16 state-of-the-art DGVMs that participated in the TRENDY (Trends and drivers of the regional scale sources and sinks of carbon dioxide) project version 11, comprising CABLE-POP, CLM5.0, CLASSIC, DLEM, IBIS, ISBA-CTRIP, ISAM, JSBACH, LPJ-GUESS, LPX-Bern, JULES, ORCHIDEE, OCN, SDGVM, VISIT-NIES, and YIBs (63). We used GPP data from the S3 simulations that are based on all time-varying forcings of CO_2_, climate, nitrogen deposition, and land use.

### Climate data

We used daily European gridded observational (E-OBS v27.0e) climate data at 0.1° spatial resolution (temperature, precipitation, and radiation) produced by ECA & D (European Climate Assessment & Dataset) project (64) in species-scale analysis, with PEP725 data to calculate temperature changes and determine temperature-relevant optimal preseason length (see Analyses), and daily Multi-Source Weather (MSWX) climate data at 0.1° spatial resolution (65) were used in the biome-scale analysis, with satellite-derived LSD; VPD was calculated using “RHtoVPD” function of “plantecophys” package in R language, based on MSWX climate data (monthly temperature, atmospheric pressure, and relative humidity) (66). We obtained the monthly SPEI dataset (three-month scale) from Consejo Superior de Investigaciones Científicas to calculate soil water availability changes (67).

### Cold degree day

We computed cold day degrees to assess climate forcing under warming and cooling (38):


(4) $$\mathrm{CD}D_{d}=\;\max\;(T_{\mathrm{base}}-T_{\mathrm{daily}},\;0)$$

(5) $$\mathrm{CDD}\;=\overset{\mathrm{LSD}}{\underset{d\;=\;d_{0}}{\sum }}\;\mathrm{CD}D_{d}$$

where $\mathrm{CD}D_{d}$ represents the CDD for date *d* and $T_{\mathrm{daily}}$ is the daily mean temperature; base temperature ($T_{\mathrm{base}}$) was set 5, 10, and 15 °C (13) and $\mathrm{CD}D_{d}=0$ when $T_{\mathrm{daily}}\;$ > $T_{\mathrm{base}}$. We computed the accumulated CDD during the period 1st July ($d_{0}$) to LSD (15), using the daily MSWX temperature dataset.

### Analyses

We analyzed ground and remotely sensed based data at forest tree species and biome scales, respectively. For ground-based observations, we utilized PEP725 LSD and E-OBS climate data to identify autumn warming and cooling samples for each tree species, where we selected the highest coefficient from a partial correlation analysis (*P* < 0.05) to determine the optimal preseason period, based on the timing of the greatest impact of temperature on LSD, whilst accounting for the effects of radiation and precipitation; temperature effects on LSD were analyzed for 8 to 120 d before mean LSD, with a step of 8 d. Then, we calculated LSD responses to temperature changes during the optimal preseason using ridge regression and multiple linear regression analyses, while controlling for effects of precipitation and radiation. For ridge regression analysis, we calculated and normalized (0–1) anomalies for each variable, prior to estimation of sensitivities. We used linear least-squares regression, utilizing year as an independent variable, to calculate trends in autumn temperature (September to November) at *P* < 0.05. Given the diverse temporal spans of ground observations, we utilized a 15-year moving window to detect autumn warming and cooling periods within each LSD time series for individual tree species; then, we calculated species average LSD responses to autumn warming and cooling, based on site mean values, and differences in response to warming and cooling were analyzed using Student's t test method at *P* < 0.05. Process framework for identifying warming and cooling samples can be found in Fig. S10.

For analysis of remotely sensed data, we applied the NH autumn cooling period (2004–2018) to detect autumn warming and cooling grid cells for forest biome types obtained from the MODIS MCD12C1 IGBP land cover product. Due to a lack of warming grid cells for evergreen broadleaf and deciduous needleleaf forests, analyses of LSD responses were restricted to evergreen needleleaf, deciduous broadleaf, and mixed forests. Differences in mean biome LSD responses between warming and cooling were analyzed utilizing Student's t test method at *P* < 0.05. We analyzed differences in LSD responses (Student's t test at *P* < 0.05) to shifts in warming and cooling between the periods 1989–2003 and 2004–2018, based on grid cells in which warming was followed by cooling or vice versa. We found no evidence for effects of day length on LSD responses to shifts in autumn temperature (Fig. 2D and Figs. S1 and S11).

We tested for drivers of shape of LSD responses to shifts in temperature, by analyzing sensitivities of LSD to CDD, frost risk, and water availability between autumn warming and cooling conditions during the period 2004–2018. Differences in the sensitivities of LSD to CDD were tested using linear mixed models (30), covariance analysis (68, 69), and random slope models (70). The linear mixed model analyses were based on linear regression analysis of site-based warming and cooling sensitivities of LSD to CDD that were then compared with direction of autumn temperature change as a fixed effect and random intercepts among forest biomes. Covariance analysis comprised a comparison of fitted regression slopes between LSD and CDD under autumn warming and cooling conditions, while a random slope model compared temporal LSD–CDD regression slopes between autumn warming and cooling conditions, with grid cell latitude and longitude as random factors (70).

We defined a frost risk index (0–1) that was standardized across the number of frost days in the studied warming and cooling grid cells, based on daily minimum temperature <0 °C during the preseason period (71, 72), and we calculated mean SPEI (three-month scale) during the preseason period. Linear mixed models were used to test for differences in frost risk and mean SPEI between autumn warming and cooling conditions.

Autumn plant productivity was estimated from mean kNDVI and accumulated GPP from September to November (26, 28) and sensitivities of autumn productivity to autumn temperature were calculated using multiple linear regressions, controlling for effects of radiation and precipitation. PLS–SEM and random forest models were used to examine effects of sensitivity of LSD to temperature S(LSD|T) on sensitivities of autumn productivity (i.e. kNDVI) to temperature S(kNDVI|T) and differences in autumn GPP sensitivities to warming and cooling estimated by 16 DGVMs were analyzed using Student's t test at *P* < 0.05. We quantified the extent to which the asymmetry GPP responses impact carbon budget, based on the asymmetry responses derived from three GPP datasets (i.e. GLASS-GPP, TLLUE-GPP, and NIRv-GPP) and the coverage of the three studied forest biomes in the NH (i.e. evergreen needleleaf, deciduous broadleaf, and mixed forests) using MODIS MCD12C1 land cover dataset (Fig. 4A and Fig. S8). The bias when not accounting for the asymmetric productivity response was calculated based on the product of the forest area and the difference in GPP responses to 1 °C warming and cooling (Fig. 4A and Fig. S8).

## Supplementary Material

pgae477_Supplementary_Data

## Data Availability

All study data are public and included in SI appendix. The specific links for data used in this study can be found in SI Appendix, Table S1. The codes used for data analysis are available on Zenodo at https://zenodo.org/doi/10.5281/zenodo.10677097.

## References

[1] Zohner CM, Renner SS, Sebald V, Crowther TW. 2021. How changes in spring and autumn phenology translate into growth-experimental evidence of asymmetric effects. J Ecol. 109:2717–2728.

[2] Keenan TF, et al 2014. Net carbon uptake has increased through warming-induced changes in temperate forest phenology. Nat Clim Change. 4:598–604.

[3] Wu C, et al 2013. Interannual variability of net ecosystem productivity in forests is explained by carbon flux phenology in autumn. Glob Ecol Biogeogr. 22:994–1006.

[4] Killingbeck KT . 1996. Nutrients in senesced leaves: keys to the search for potential resorption and resorption proficiency. Ecology 77:1716–1727.

[5] Estiarte M, Peñuelas J. 2015. Alteration of the phenology of leaf senescence and fall in winter deciduous species by climate change: effects on nutrient proficiency. Glob Change Biol. 21:1005–1017. 10.1111/gcb.12804 25384459

[6] Jeong S . 2020. Autumn greening in a warming climate. Nat Clim Change. 10:712–713.

[7] Jeong S-J, Ho C-H, Gim H-J, Brown ME. 2011. Phenology shifts at start vs. end of growing season in temperate vegetation over the Northern Hemisphere for the period 1982-2008. Glob Change Biol. 17:2385–2399.

[8] Zani D, Crowther TW, Mo L, Renner SS, Zohner CM. 2020. Increased growing-season productivity drives earlier autumn leaf senescence in temperate trees. Science 370:1066–1071. 33243884 10.1126/science.abd8911

[9] Gill AL, et al 2015. Changes in autumn senescence in northern hemisphere deciduous trees: a meta-analysis of autumn phenology studies. Ann Bot. 116:875–888. 25968905 10.1093/aob/mcv055 PMC4640124

[10] Gallinat AS, Primack RB, Wagner DL. 2015. Autumn, the neglected season in climate change research. Trends Ecol Evol. 30:169–176. 25662784 10.1016/j.tree.2015.01.004

[11] Walther G-R, et al 2002. Ecological responses to recent climate change. Nature 416:389–395. 11919621 10.1038/416389a

[12] Piao S, et al 2019. Plant phenology and global climate change: current progresses and challenges. Glob Change Biol. 25:1922–1940. 10.1111/gcb.14619 30884039

[13] Wu C, et al 2022. Increased drought effects on the phenology of autumn leaf senescence. Nat Clim Change. 12:943–949.

[14] Wu C, et al 2018. Contrasting responses of autumn-leaf senescence to daytime and night-time warming. Nat Clim Change. 8:1092–1096.

[15] Wu C, et al 2021. Widespread decline in winds delayed autumn foliar senescence over high latitudes. Proc Natl Acad Sci U S A. 118:e2015821118. 33846246 10.1073/pnas.2015821118 PMC8072329

[16] M. Shen, et al, Plant phenology changes and drivers on the Qinghai–Tibetan Plateau. Nat Rev Earth Environ. 3, 633–651 (2022).

[17] Ettinger AK, Buonaiuto DM, Chamberlain CJ, Morales-Castilla I, Wolkovich EM. 2021. Spatial and temporal shifts in photoperiod with climate change. New Phytol. 230:462–474. 33421152 10.1111/nph.17172

[18] Fu YSH, et al 2014. Variation in leaf flushing date influences autumnal senescence and next year's flushing date in two temperate tree species. Proc Natl Acad Sci U S A. 111:7355–7360. 24799708 10.1073/pnas.1321727111 PMC4034254

[19] Keenan TF, Richardson AD. 2015. The timing of autumn senescence is affected by the timing of spring phenology: implications for predictive models. Glob Change Biol. 21:2634–2641. 10.1111/gcb.12890 25662890

[20] Zohner CM, et al 2023. Effect of climate warming on the timing of autumn leaf senescence reverses after the summer solstice. Science 381:eadf5098. 37410847 10.1126/science.adf5098

[21] Keskitalo J, Bergquist G, Gardeström P, Jansson S. 2005. A cellular timetable of autumn senescence. Plant Physiol. 139:1635–1648. 16299183 10.1104/pp.105.066845 PMC1310548

[22] Zhao Y, et al 2016. ABA receptor PYL9 promotes drought resistance and leaf senescence. Proc Natl Acad Sci U S A. 113:1949–1954. 26831097 10.1073/pnas.1522840113 PMC4763734

[23] Woo HR, Kim HJ, Lim PO, Nam HG. 2019. Leaf senescence: systems and dynamics aspects. Annu Rev Plant Biol. 70:347–376. 30811218 10.1146/annurev-arplant-050718-095859

[24] Fu YH, et al 2022. Soil moisture regulates warming responses of autumn photosynthetic transition dates in subtropical forests. Glob Change Biol. 28:4935–4946. 10.1111/gcb.16227 35642473

[25] Samaniego L, et al 2018. Anthropogenic warming exacerbates European soil moisture droughts. Nat Clim Change. 8:421–426.

[26] Tang R, et al 2022. Increasing terrestrial ecosystem carbon release in response to autumn cooling and warming. Nat Clim Change. 12:380–385.

[27] Ballantyne A, et al 2017. Accelerating net terrestrial carbon uptake during the warming hiatus due to reduced respiration. Nat Clim Change. 7:148–152.

[28] Wang X, et al 2019. No trends in spring and autumn phenology during the global warming hiatus. Nat Commun. 10:2389. 31160586 10.1038/s41467-019-10235-8 PMC6546754

[29] He L, et al 2023. Non-symmetric responses of leaf onset date to natural warming and cooling in northern ecosystems. PNAS Nexus. 2:pgad308. 37780232 10.1093/pnasnexus/pgad308 PMC10538477

[30] Chen L, et al 2020. Leaf senescence exhibits stronger climatic responses during warm than during cold autumns. Nat Clim Change. 10:777–780.

[31] Fu YH, et al 2018. Larger temperature response of autumn leaf senescence than spring leaf-out phenology. Glob Change Biol. 24:2159–2168. 10.1111/gcb.14021 29245174

[32] Wang S, et al 2023. Larger responses of trees' leaf senescence to cooling than warming: results from a climate manipulation experiment. Agric For Meteorol. 339:109568.

[33] Li B, Li Y, Chen Y, Zhang B, Shi X. 2020. Recent fall Eurasian cooling linked to North Pacific sea surface temperatures and a strengthening Siberian high. Nat Commun. 11:5202. 33060590 10.1038/s41467-020-19014-2 PMC7567785

[34] Templ B, et al 2018. Pan European phenological database (PEP725): a single point of access for European data. Int J Biometeorol. 62:1109–1113. 29455297 10.1007/s00484-018-1512-8

[35] Li X, et al 2016. Responses of sequential and hierarchical phenological events to warming and cooling in alpine meadows. Nat Commun. 7:12489. 27535205 10.1038/ncomms12489 PMC4992149

[36] Zavaleta ES, Shaw MR, Chiariello NR, Mooney HA, Field CB. 2003. Additive effects of simulated climate changes, elevated CO_2_, and nitrogen deposition on grassland diversity. Proc Natl Acad Sci U S A. 100:7650–7654. 12810960 10.1073/pnas.0932734100 PMC164642

[37] Wolkovich EM, et al 2012. Warming experiments underpredict plant phenological responses to climate change. Nature 485:494–497. 22622576 10.1038/nature11014

[38] Delpierre N, et al 2009. Modelling interannual and spatial variability of leaf senescence for three deciduous tree species in France. Agric For Meteorol. 149:938–948.

[39] Fu YH, et al 2015. Declining global warming effects on the phenology of spring leaf unfolding. Nature 526:104–107. 26416746 10.1038/nature15402

[40] Gao M, et al 2020. Three-dimensional change in temperature sensitivity of northern vegetation phenology. Glob Change Biol. 26:5189–5201. 10.1111/gcb.15200 32475002

[41] Vitasse Y, Lenz A, Körner C. 2014. The interaction between freezing tolerance and phenology in temperate deciduous trees. Front Plant Sci. 5:541. 25346748 10.3389/fpls.2014.00541 PMC4192447

[42] Wang T, et al 2014. The influence of local spring temperature variance on temperature sensitivity of spring phenology. Glob Change Biol. 20:1473–1480. 10.1111/gcb.12509 24357518

[43] Tezara W, Mitchell VJ, Driscoll SD, Lawlor DW. 1999. Water stress inhibits plant photosynthesis by decreasing coupling factor and ATP. Nature 401:914–917.

[44] Buckley TN . 2019. How do stomata respond to water status? New Phytol. 224:21–36. 31069803 10.1111/nph.15899

[45] Tiwari RK, et al 2021. Mechanistic insights on melatonin-mediated drought stress mitigation in plants. Physiol Plant. 172:1212–1226. 33305363 10.1111/ppl.13307

[46] Eyring V, et al 2021. Human influence on the climate system. In climate change 2021: the physical science basis. Contribution of working group I to the sixth assessment report of the intergovernmental panel on climate change. IPCC Sixth Assess Rep.

[47] Sherwood S, Fu Q. 2014. A drier future? Science 343:737–739. 24531959 10.1126/science.1247620

[48] Zaitchik BF, Rodell M, Biasutti M, Seneviratne SI. 2023. Wetting and drying trends under climate change. Nat Water. 1:502–513.

[49] Seddon AWR, Macias-Fauria M, Long PR, Benz D, Willis KJ. 2016. Sensitivity of global terrestrial ecosystems to climate variability. Nature 531:229–232. 26886790 10.1038/nature16986

[50] Wu X, et al 2017. Higher temperature variability reduces temperature sensitivity of vegetation growth in Northern Hemisphere. Geophys Res Lett. 44:6173–6181.

[51] Sun Z, et al 2019. Evaluating and comparing remote sensing terrestrial GPP models for their response to climate variability and CO_2_ trends. Sci Total Environ. 668:696–713. 30856578 10.1016/j.scitotenv.2019.03.025

[52] Lapola DM, et al 2023. The drivers and impacts of Amazon forest degradation. Science 379:eabp8622. 36701452 10.1126/science.abp8622

[53] Shen M, et al 2020. Can changes in autumn phenology facilitate earlier green-up date of northern vegetation? Agric For Meteorol. 291:108077.

[54] Wang J, Liu D, Ciais P, Peñuelas J. 2022. Decreasing rainfall frequency contributes to earlier leaf onset in northern ecosystems. Nat Clim Change. 12:386–392.

[55] Zhang X, et al 2003. Monitoring vegetation phenology using MODIS. Remote Sens Environ. 84:471–475.

[56] Gonsamo A, Chen JM, Price DT, Kurz WA, Wu C. 2012. Land surface phenology from optical satellite measurement and CO_2_ eddy covariance technique. J. Geophys Res Biogeosci. 117:2012JG002070.

[57] White MA, Thornton PE, Running SW. 1997. A continental phenology model for monitoring vegetation responses to interannual climatic variability. Glob Biogeochem Cycles. 11:217–234.

[58] Camps-Valls G, et al 2021. A unified vegetation index for quantifying the terrestrial biosphere. Sci Adv. 7:eabc7447. 33637524 10.1126/sciadv.abc7447 PMC7909876

[59] Didan K . 2021. MODIS/terra vegetation indices monthly L3 global 0.05 Deg CMG V061. NASA EOSDIS Land Process. DAAC. 61. 10.5067/MODIS/MOD13C2.061.

[60] Liang S, et al 2021. The global land surface satellite (GLASS) product suite. Bull Am Meteorol Soc. 102:E323–E337.

[61] Bi W, et al 2022. A global 0.05° dataset for gross primary production of sunlit and shaded vegetation canopies from 1992 to 2020. Sci Data. 9:213. 35577806 10.1038/s41597-022-01309-2 PMC9110750

[62] Wang S, Zhang Y, Ju W, Qiu B, Zhang Z. 2021. Tracking the seasonal and inter-annual variations of global gross primary production during last four decades using satellite near-infrared reflectance data. Sci Total Environ. 755:142569. 33038811 10.1016/j.scitotenv.2020.142569

[63] Friedlingstein P, et al 2022. Global carbon budget 2022. Earth Syst Sci Data. 14:4811–4900.

[64] Cornes RC, Van Der Schrier G, Van Den Besselaar EJM, Jones PD. 2018. An ensemble version of the E-OBS temperature and precipitation data sets. J Geophys Res Atmosph. 123:9391–9409.

[65] Beck HE, et al 2022. Global 3-hourly 0.1° bias-corrected meteorological data including near-real-time updates and forecast ensembles. Bull Am Meteorol Soc. 103:E710–E732.

[66] Duursma RA . 2015. Plantecophys—an R package for analysing and modelling leaf gas exchange data. PLoS One. 10:e0143346. 26581080 10.1371/journal.pone.0143346 PMC4651500

[67] Vicente-Serrano SM, Beguería S, López-Moreno JI. 2010. A multiscalar drought Index sensitive to global warming: the standardized precipitation evapotranspiration Index. J Clim. 23:1696–1718.

[68] Zar JH . 2010. Biostatistical analysis. 5th ed. Prentice-Hall/Pearson.

[69] He L, Li Z-L, Wang X, Xie Y, Ye J-S. 2021. Lagged precipitation effect on plant productivity is influenced collectively by climate and edaphic factors in drylands. Sci Total Environ. 755:142506. 33035982 10.1016/j.scitotenv.2020.142506

[70] Bates D, Mächler M, Bolker B, Walker S. 2015. Fitting linear mixed-effects models using lme4. J Stat Softw. 67(1):1–48.

[71] Easterling DR . 2002. Recent changes in frost days and the frost-free season in the United States. Bull Am Meteorol Soc. 83:1327–1332.

[72] Meehl GA, Tebaldi C, Nychka D. 2004. Changes in frost days in simulations of twentyfirst century climate. Clim Dyn. 23:495–511.

