# Supplementary material for: Asymmetric temperature effect on leaf senescence and its control on ecosystem productivity
Source: PNAS Nexus. 2024 Oct 23;3(11):pgae477. doi: 10.1093/pnasnexus/pgae477 (PMC11529893; doi:10.1093/pnasnexus/pgae477)
Supplement: pgae477_Supplementary_Data [file pgae477_supplementary_data.docx]

Supplementary Information (SI) Appendix for:

**Asymmetric temperature effect on leaf senescence and its control on ecosystem productivity**

**This PDF file includes:**

**Supplementary Tables and Figures**

Table S1

Figs. S1 to S11

Table S1. Details and sources of data used in this study.

| Data type | Data name | Unit | Spatial resolution | Temporal resolution | Spatial | Source |
| --- | --- | --- | --- | --- | --- | --- |
|  |  |  |  |  | Range |  |
| LSD | Leaf senescence | DOY | - | yearly | Local | PEP725^1^ |
| Greenness | NDVI | - | 1/12° | bi-weekly | Global | PKU GIMMS^2^ |
| Climate data | Temperature | °C | 0.1° | daily | Global | MSWX^3^ |
|  | Precipitation | mm | 0.1° | daily | Global | MSWX |
|  | Solar radiation | W m^-2^ | 0.1° | daily | Global | MSWX |
|  | Atmospheric pressure | Pa | 0.1° | monthly | Global | MSWX |
|  | Relative humidity | % | 0.1° | monthly | Global | MSWX |
|  | Temperature | °C | 0.1° | monthly | Global | MSWX |
|  | Temperature | °C | 0.1° | daily | Europe | E-OBS^4^ |
|  | Precipitation | mm | 0.1° | daily | Europe | E-OBS |
|  | Solar radiation | W m^-2^ | 0.1° | daily | Europe | E-OBS |
|  | SPEI | - | 0.5° | monthly | Global | CSIC^5^ |
| Plant productivity | kNDVI | - | 0.05° | monthly | Global | MOD13C2^6^ |
|  | GPP | g C m^-2^ mon^-1^ | 0.05° | monthly | Global | NIRv GPP^7^ |
|  | GPP | g C m^-2^ mon^-1^ | 0.05° | monthly | Global | GLASS GPP^8^ |
|  | GPP | g C m^-2^ mon^-1^ | 0.05° | monthly | Global | TL-LUE GPP^9^ |
|  | GPP | g C m^-2^ mon^-1^ | 0.5° | monthly | Global | TRENDY v11^10^ |
| Land cover | Land cover | - | 0.05° | yearly | Global | MCD12C1^11^ |

^1^ <http://www.pep725.eu/index.php>

^2^ <https://zenodo.org/records/8253971>

^3^ <https://www.gloh2o.org/mswx>

^4^ <https://www.ecad.eu/download/ensembles/download.php>

^5^ <https://spei.csic.es/database.html>

^6^ <https://lpdaac.usgs.gov/products/mod13c2v061>

^7^ <https://doi.org/10.6084/m9.figshare.12981977.v2>

^8^ <http://www.glass.umd.edu/Download.html>

^9^ <https://doi.org/10.5061/dryad.dfn2z352k>

^10^ [https://blogs.exeter.ac.uk/trendy](https://blogs.exeter.ac.uk/trendy/)

^11^ <https://lpdaac.usgs.gov/products/mcd12c1v006>


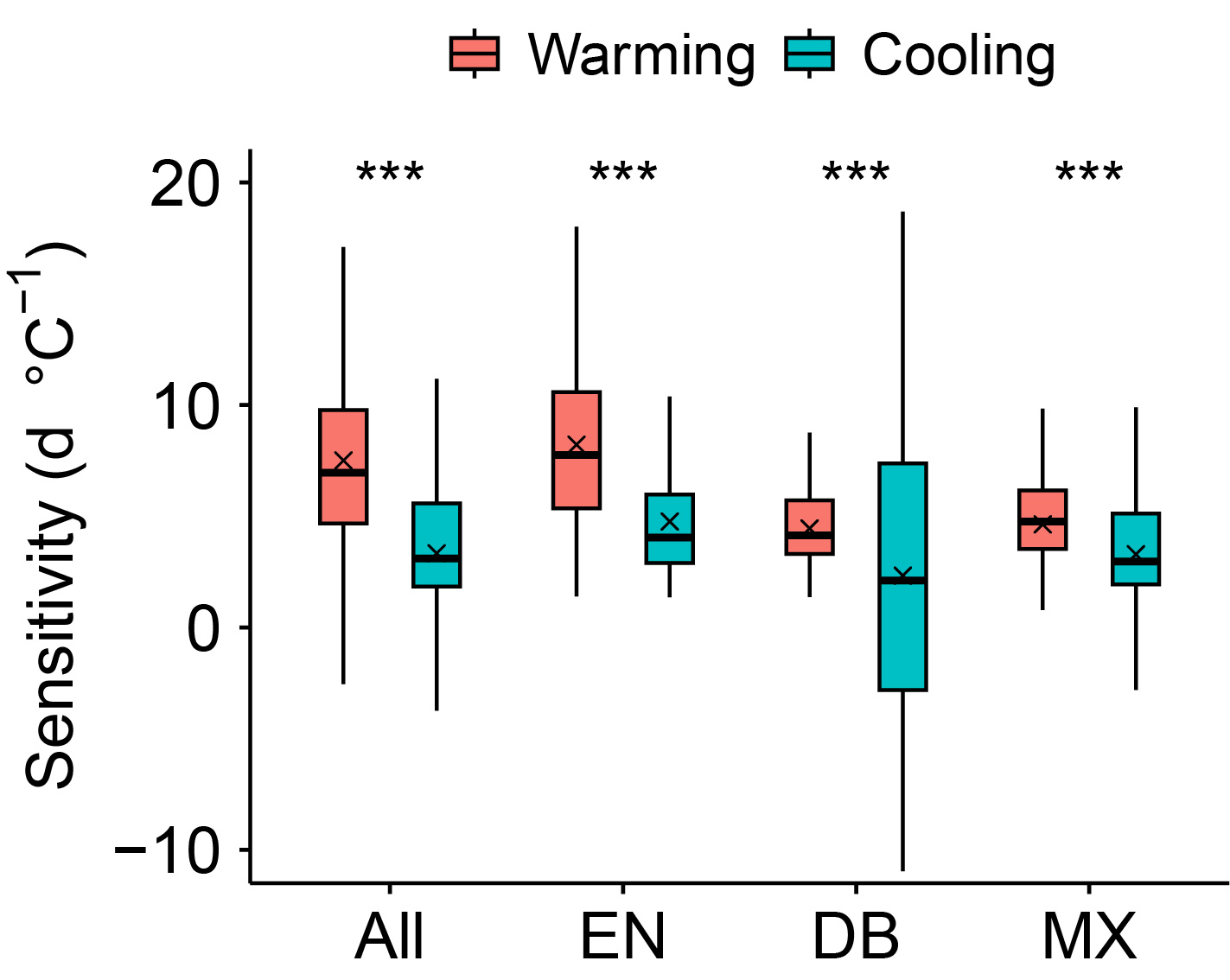


**Fig. S1.** Responses of leaf senescence date (LSD) to autumn warming and cooling in evergreen needleleaf (EN), deciduous broadleaf (DB), and mixed forests (MX) during the period 2004–2018, based on multiple linear regression analysis. Differences in LSD responses between warming and cooling conditions were analyzed using Student's t-test at *P* < 0.05. Boxplots show median (horizontal line) and mean (cross) data within the 25–75th percentiles; ****P* < 0.001.


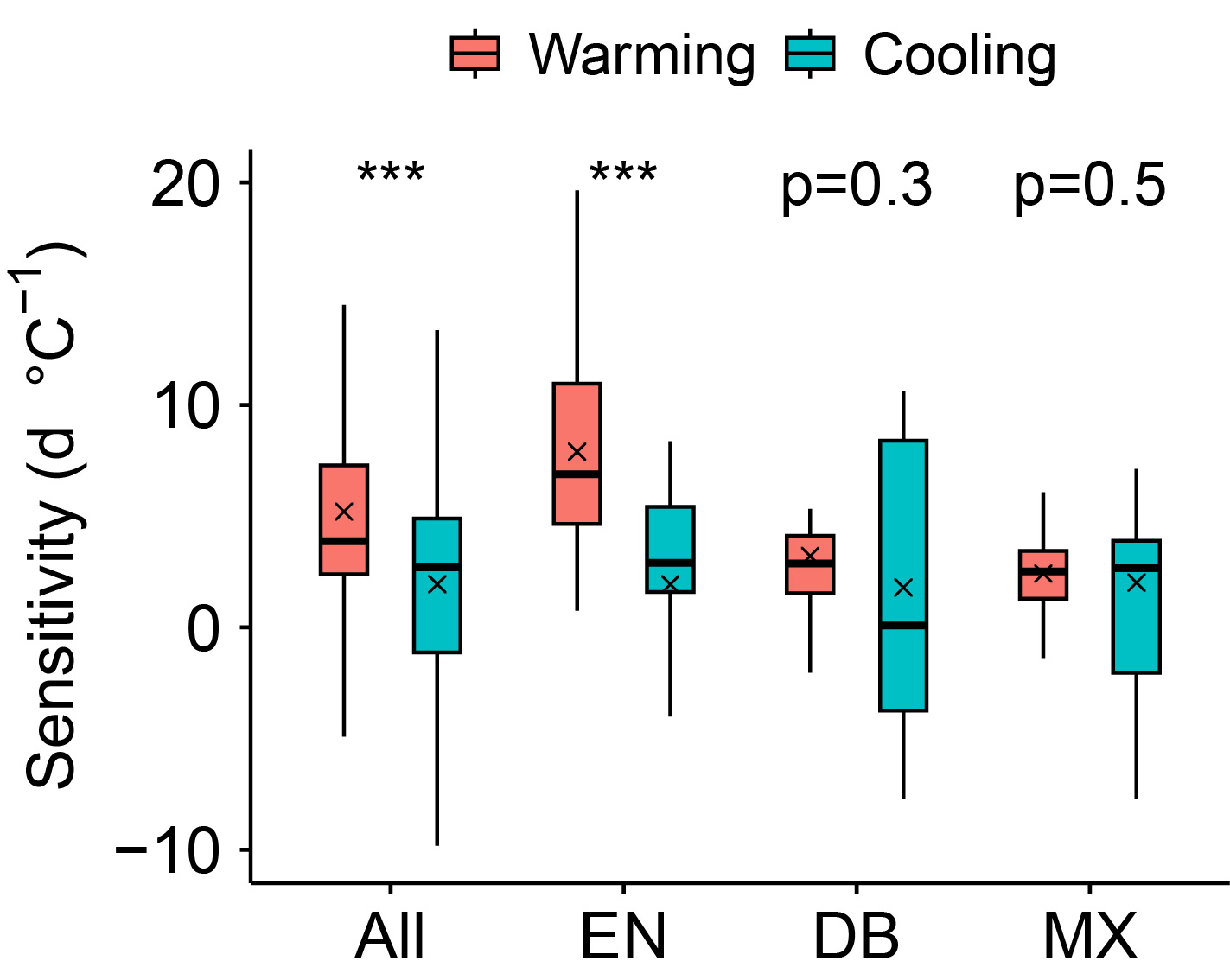


**Fig. S2.** LSD responses to shifts in autumn warming and cooling between the periods 1989–2003 and 2004–2018 in evergreen needleleaf (EN), deciduous broadleaf (DB), and mixed forests (MX), based on multiple linear regression analysis. Differences in LSD responses between warming and cooling conditions were analyzed using Student's t-test at *P* < 0.05. Boxplots show median (horizontal line) and mean (cross) data within the 25–75th percentiles; ****P* < 0.001.


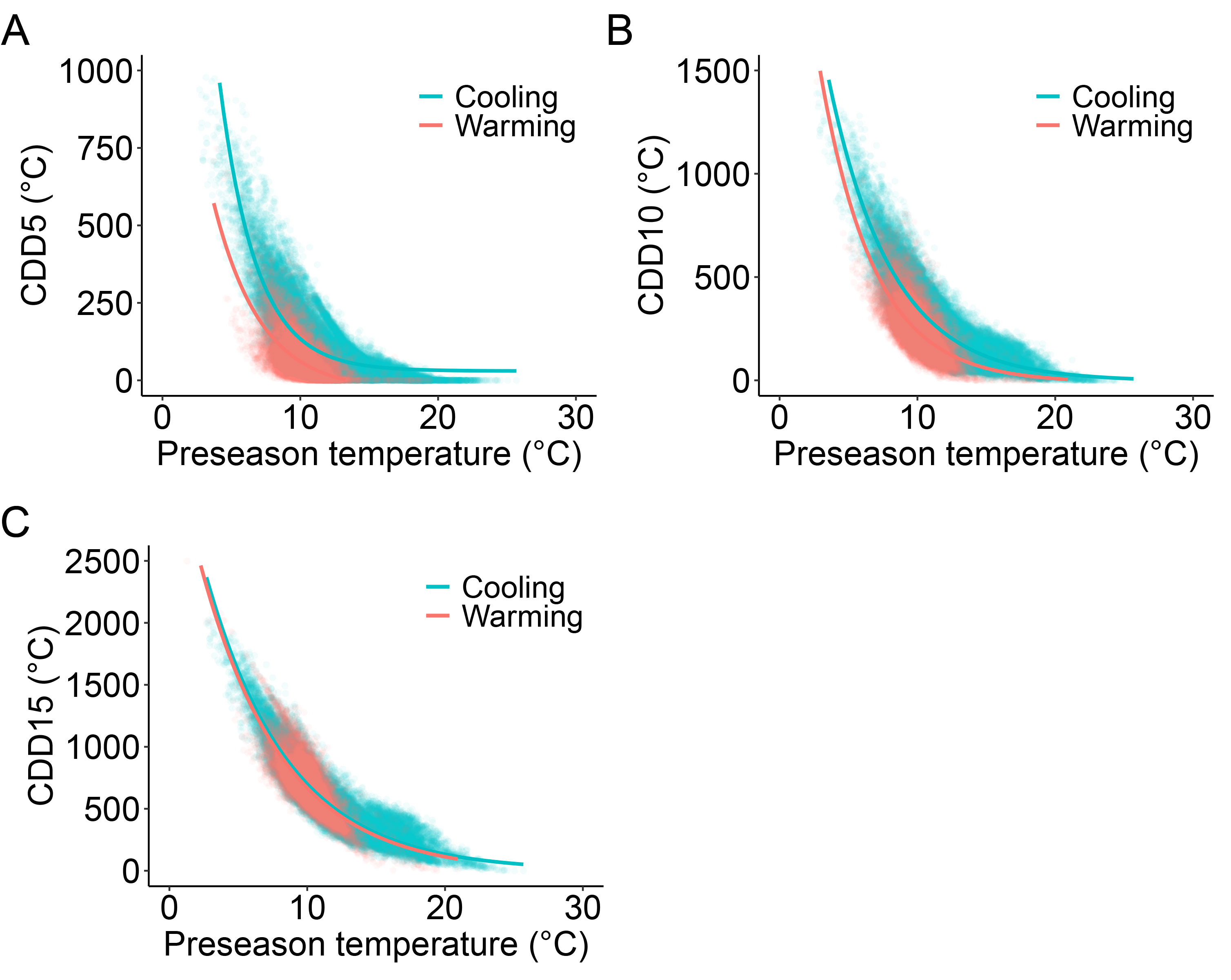


**Fig. S3.** The relationship between cold degree days (CDD) and preseason temperature with CDD base temperatures of **A)** 5°C, **B**) 10°C, and **C**) 15°C.


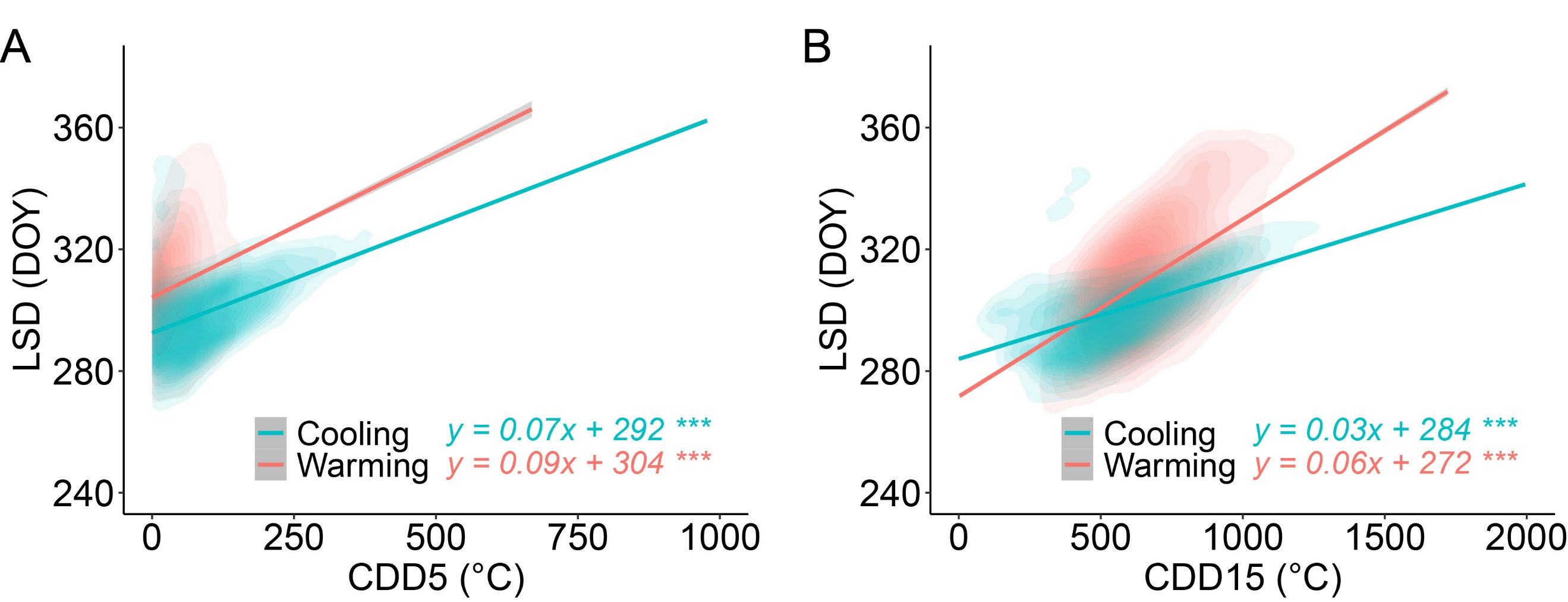


**Fig. S4.** Comparison of relations between LSD and CDD under autumn warming and cooling conditions, with CDD base temperatures of **A**) 5°C and **B**) 15°C.


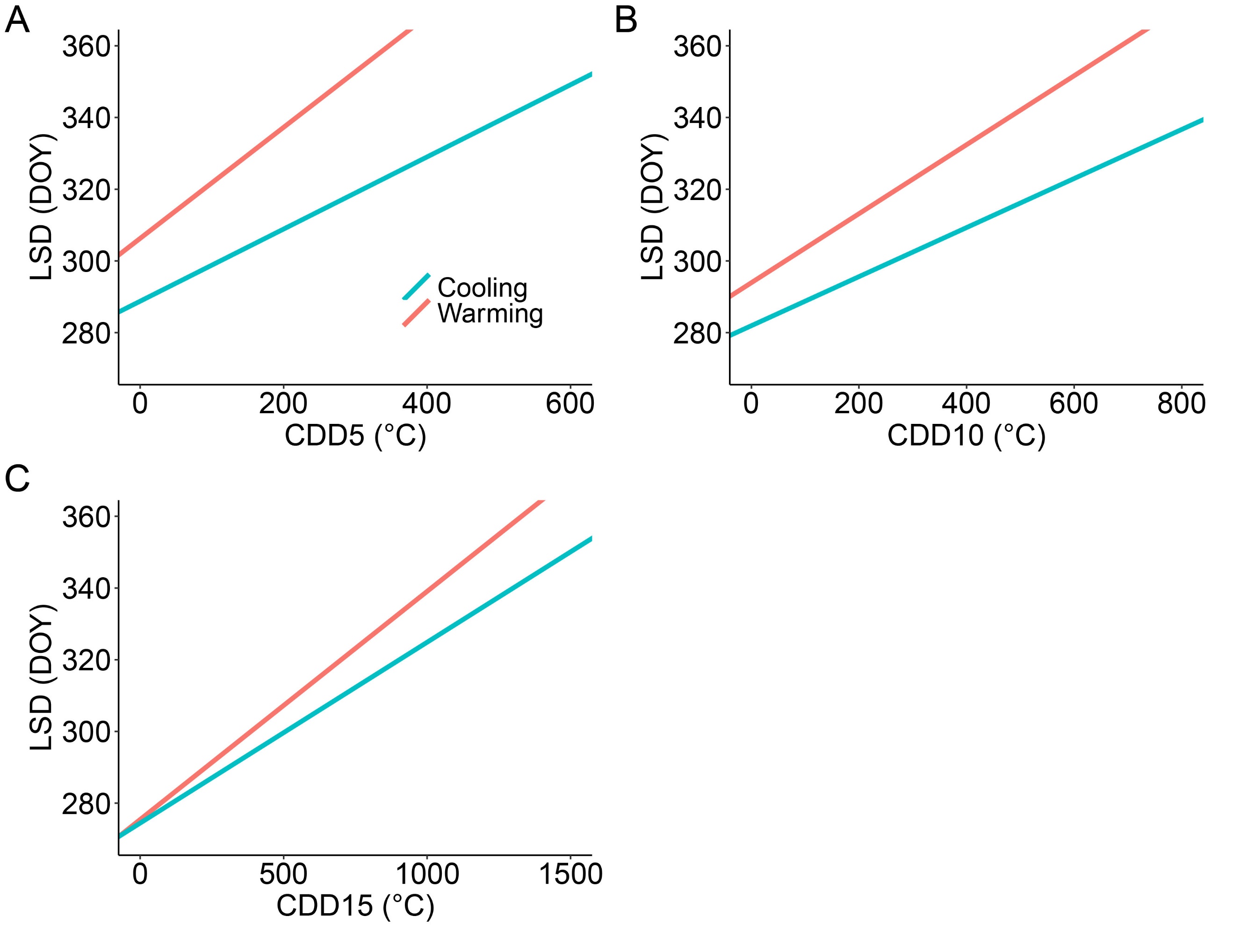


**Fig. S5.** Random slope model comparisons of relations between LSD and CDD under autumn warming and cooling conditions, with CCD base temperatures of **A**) 5°C, **B**) 10°C, and **C**) 15°C.


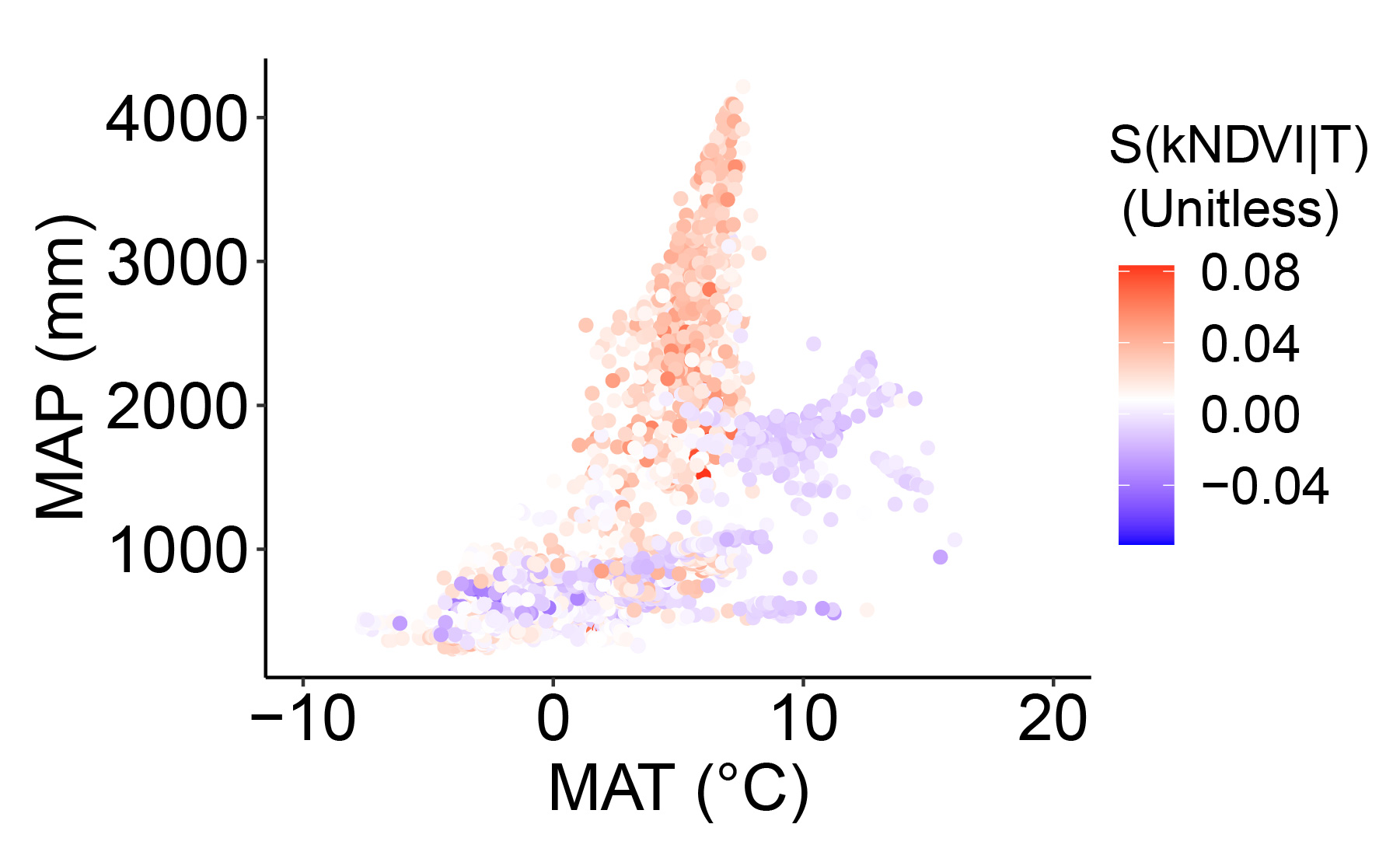


**Fig. S6.** Study site productivity (kNDVI) sensitivities to climate space of mean annual precipitation (MAP) and mean annual temperature (MAT).


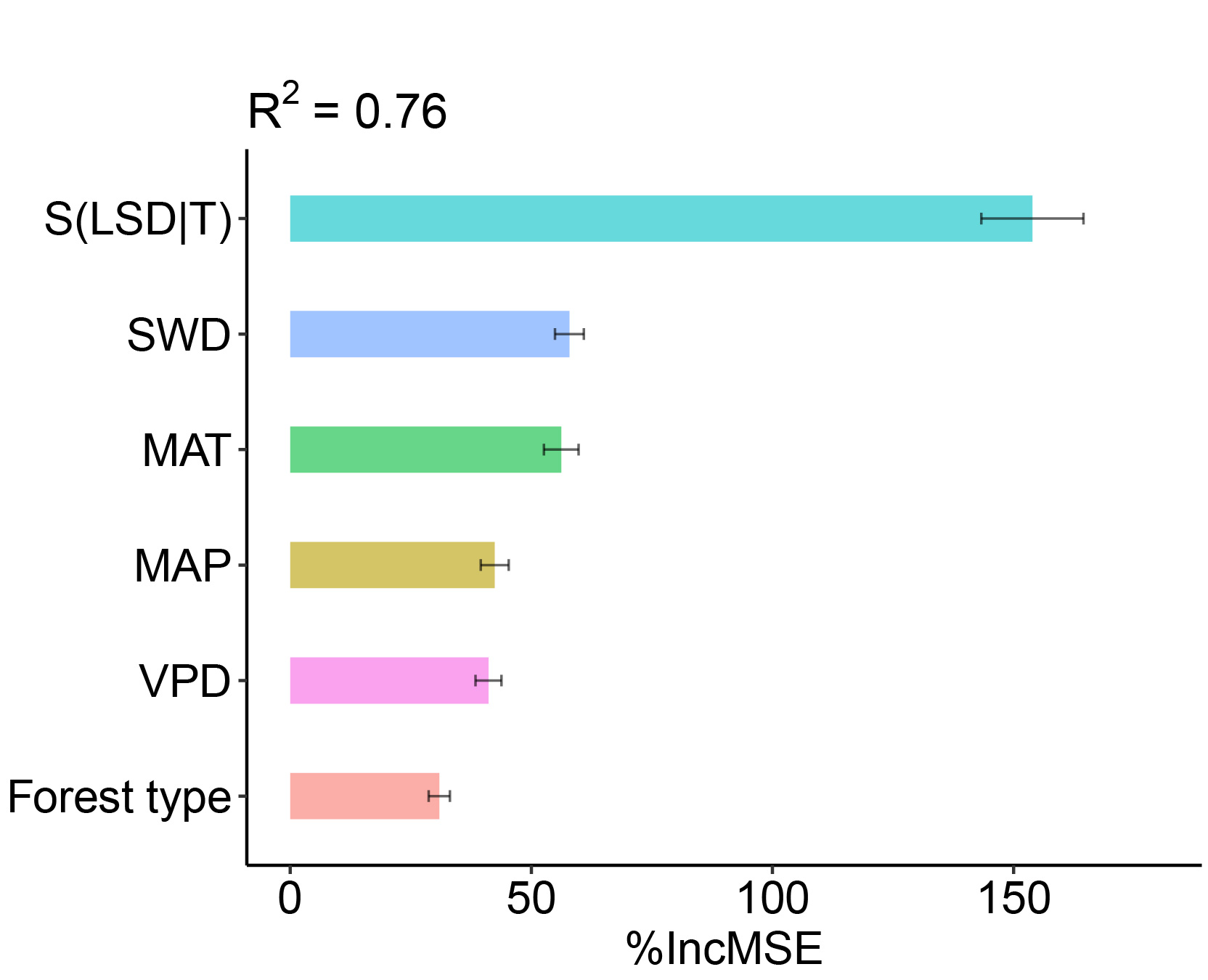


**Fig. S7.** Random forest analysis of mean relative importance of predictors of autumn productivity sensitivities (kNDVI) to temperature, based on percent increase in mean squared error (%IncMSE). S(LSD|T): LSD sensitivity to temperature; SWD: downward solar radiation; MAT: mean annual temperature; MAP: mean annual precipitation; and, VPD: vapor pressure deficit. Error bars are mean SEs of 1,000 bootstrap samples.


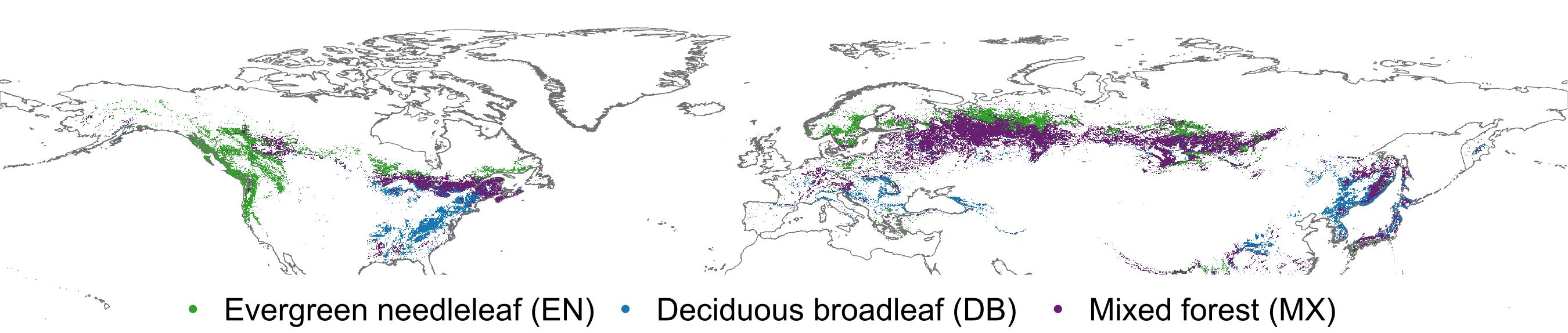


**Fig. S8.** The spatial distribution of evergreen needleleaf, deciduous broadleaf, and mixed forest in 2018 in the northern hemisphere.


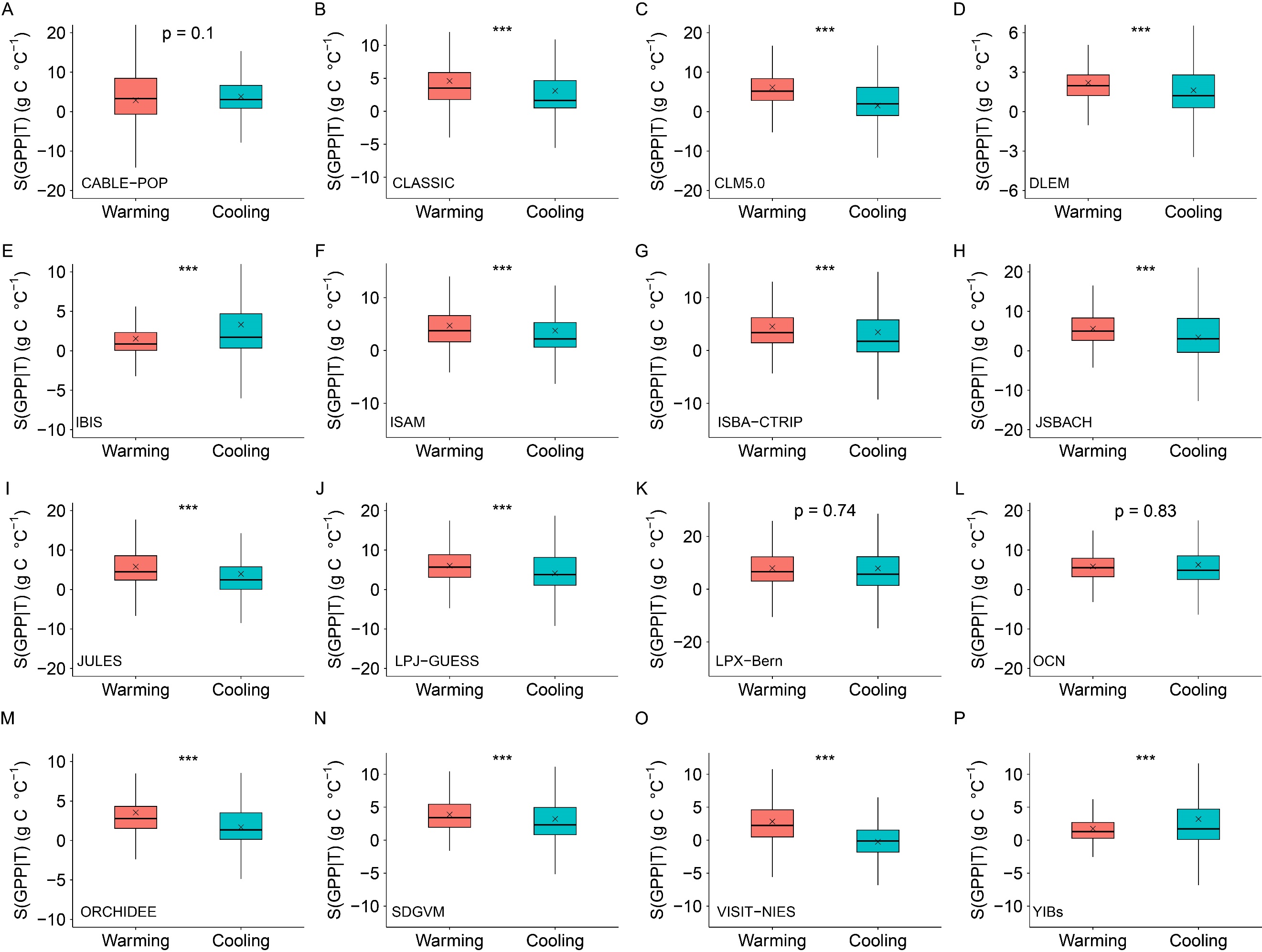


**Fig. S9.** Estimated sensitivities of autumn GPP to temperature under autumn warming and cooling conditions in 16 state-of-the-art Dynamic Global Vegetation Models. Differences in sensitivities to autumn warming and cooling were tested using a linear mixed model, with random intercepts among forest biomes. Boxplots show median (horizontal line) and mean (cross) data within the 25–75th percentiles; ****P* < 0.001.


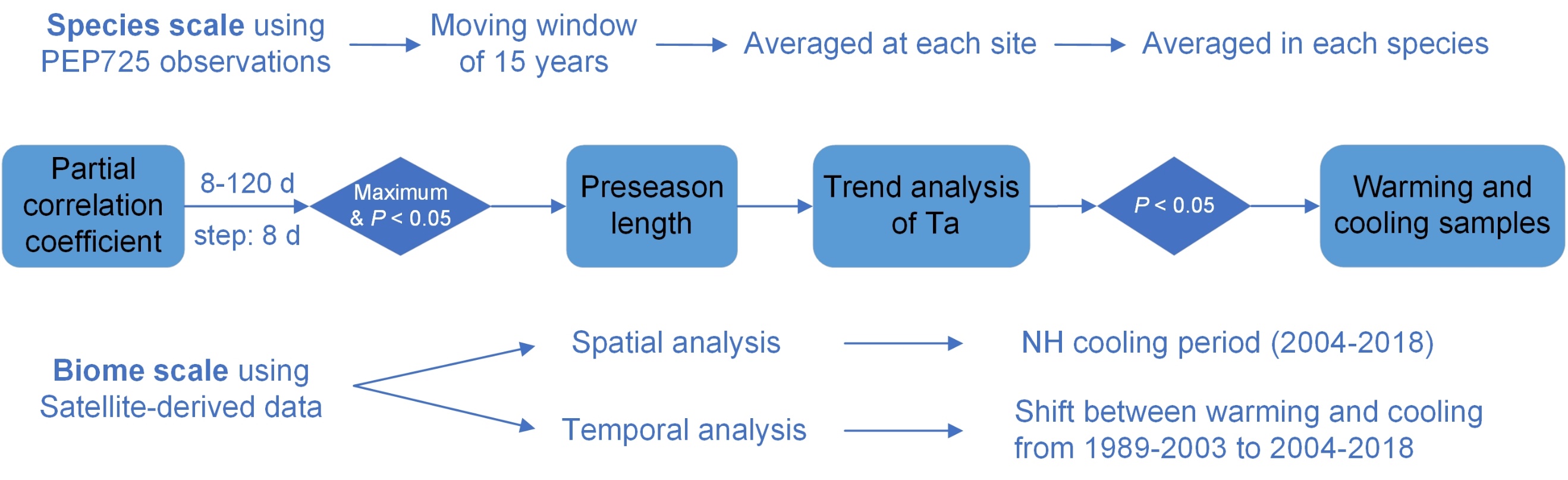


**Fig. S10.** The process framework for identifying warming and cooling samples at the species and biome scale.


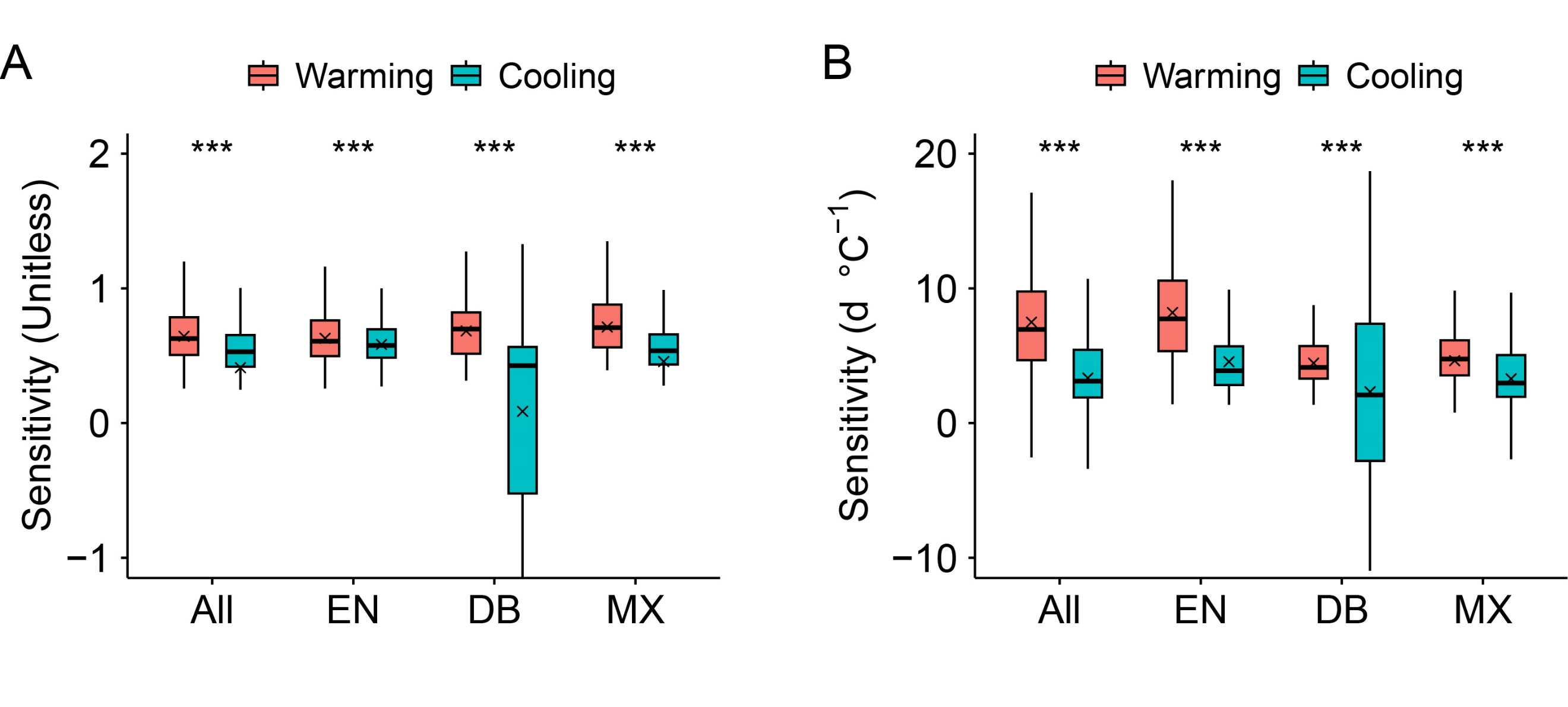


**Fig. S11.** Ridge regression (**A**) and multiple linear regression (**B**) analyses of forest biome LSD sensitivities to autumn warming and cooling during the period 2004–2018, controlling for effects of day length. EN: among evergreen needleleaf, DB: deciduous broadleaf, and MX: mixed forests. Differences in LSD responses between warming and cooling conditions were analyzed using Student's t-test at *P* < 0.05. Boxplots show median (horizontal line) and mean (cross) data within the 25–75th percentiles; ****P* < 0.001.
